# Gut Microbiota of Captive and Wild Siberian Cranes and Links to Soil in Poyang Lake Wetlands

**DOI:** 10.3390/ani16060894

**Published:** 2026-03-12

**Authors:** Zheng Lai, Liting Xiao, Huilin Yang, Wenjing Yang, Qinghui You, Chaosheng Zhang, Minfei Jian

**Affiliations:** 1 Key Laboratory of Biodiversity Conservation and Bioresource Utilization of Jiangxi Province, College of Geography and Environment, Jiangxi Normal University, Nanchang 330022, China; laizheng@fosu.edu.cn (Z.L.); wenjing0282@163.com (W.Y.); 2 College of Life Sciences, Jiangxi Normal University, Nanchang 330022, China; xiaolt014726@163.com (L.X.); yanghuilin123@163.com (H.Y.); qinghuiyou@jxnu.edu.cn (Q.Y.); 3 Key Laboratory of Poyang Lake Wetlands and Watershed Research, Ministry of Education, Jiangxi Normal University, Nanchang 330022, China; 4 School of Medicine, Foshan University, Foshan 528225, China; 5 International Network for Environment and Health (INEH), School of Geography, Archaeology and Irish Studies, Ryan Institute, University of Galway, H91 CF50 Galway, Ireland; chaosheng.zhang@nuigalway.ie

**Keywords:** Siberian cranes, gut microbiota, soil microbiota, high-throughput sequencing

## Abstract

Gut microbiota are integral to host health and ecological adaptation, yet their interactions with environmental microbial communities remain understudied in migratory waterbirds. Using high-throughput 16S rRNA gene sequencing, we compared gut microbiota of captive and wild Siberian cranes and their associations with soil microbiota in the Poyang Lake wetlands. Alpha diversity was significantly higher in soil than in gut microbiota, with captive cranes exhibiting greater microbial richness and evenness than wild individuals. Beta diversity analysis revealed distinct gut and soil microbiota, with partial overlap between captive and wild crane gut microbiota. Firmicutes dominated gut communities, with *Ligilactobacillus* and *Romboutsia* enriched in captive cranes, whereas *Acidobacteria* were predominant in soil. *Escherichia*-*Shigella* was more abundant in wild cranes and soil. Linear discriminant analysis (LDA) effect size (LEfSe) analysis identified 34 differentially enriched taxa, and microbial network analysis indicated stronger gut–soil microbial associations than those between captive and wild hosts. Network analysis further revealed distinct co-occurrence patterns between captive and wild groups, suggesting potential shifts in microbial interaction structures under different living conditions. These findings provide preliminary insights that may inform future conservation strategies for Siberian cranes.

## Simple Summary

The gut microbiota of captive and wild Siberian cranes (*Leucogeranus leucogeranus*) and their associations with soil microbiota in the Poyang Lake wetlands were analyzed and compared in this study. Captive cranes had richer and more even gut microbiota communities than their wild counterparts. While gut and soil microbiota were distinct overall, the gut communities of captive and wild cranes showed some similarity. Firmicutes was the dominant gut microbiota group. Notably, captive cranes had higher levels of certain microbiota like *Ligilactobacillus*, while wild cranes and soil samples showed higher levels of *Escherichia*-*Shigella*. Crucially, the analysis revealed compositional similarities between soil and gut samples. This knowledge can help inform better management and conservation strategies for the Siberian cranes.

## 1. Introduction

Gut microbiota play a vital role in host health and physiology, contributing to digestion, metabolism, immune homeostasis, and overall fitness [1,2]. Their composition and diversity are shaped by intrinsic (genetics, immunity, age) and extrinsic factors (diet, habitat, environmental microbiota) [3,4,5,6]. Understanding these microbial communities is crucial for assessing ecological adaptations and conservation strategies for endangered species [3]. Captive breeding is essential for species conservation but imposes significant environmental shifts that can alter gut microbiota [7,8]. Factors such as controlled diets, reduced microbial exposure, antibiotic use, and human interactions may lead to lower microbial diversity, increased opportunistic pathogens, and functional impairments in digestion and immunity [4,9]. While some studies suggest captivity may enhance immune status, its overall impact remains debated [10]. Thus, research on gut microbiota can improve survival of animals in different living environments [11]. Evaluating gut microbiota differences between captive and wild individuals is critical for optimizing conservation management.

The Siberian crane was classified as the critically endangered (CR) crane species by the International Union for Conservation of Nature (IUCN) and faces serious threats from habitat degradation and climate change [12]. Currently, the global population of Siberian cranes is estimated to be approximately 3500–4000 individuals. Among these, Poyang Lake wetlands serve as the most critical wintering habitat, supporting over 98% of the global population [13]. With Poyang Lake wetlands as its key wintering ground, conservation efforts have focused on captive breeding and reintroduction. However, microbiota shifts in captivity may influence health and adaptability, potentially affecting reintroduction success. Despite studies on Siberian crane diet, behavior, and gut microbiota composition [14,15,16,17], the role of environmental microbiota, particularly soil microbes, remains unclear.

As a vast microbial reservoir, soil plays a fundamental role in shaping gut microbiota through feeding, foraging, and excretion [18,19,20]. Microbial exchange between soil and gut communities can influence host digestion, metabolism, and immunity [21,22], yet this interaction remains understudied in avian conservation.

Our previous research [17] primarily characterized the compositional structure of the gut microbiota in wintering Siberian cranes. Building upon this foundation, the present study shifts the focus from a descriptive analysis to the exploratory investigation of the associations between the gut microbiota and the soil microbiota of the habitat. This study aims to: (1) characterize gut microbiota differences between captive and wild Siberian cranes for first time, (2) analyze soil microbiota composition in their respective habitats, and (3) explore gut–soil microbial associations to assess environmental influences on gut microbiota. This exploratory study aims to investigate whether differences exist in gut microbiota composition between captive and wild Siberian cranes and whether habitat conditions are associated with soil microbial community composition. Using 16S rRNA high-throughput sequencing, this study describes the gut and soil microbial communities associated with captive and wild Siberian cranes, offering a reference for future rewilding and conservation research.

To ensure the validity of comparisons between captive and wild populations, the sampling sites were carefully selected to minimize confounding environmental background interference. The sampling sites are located within the same climatic zone and share comparable geographical features. This geographical proximity and climatic consistency help to ensure that any observed differences in gut and soil microbiota between captive and wild cranes can be more reliably attributed to captivity-related factors rather than to large-scale climatic or geographical variations.

## 2. Materials and Methods

### 2.1. Study Area and Sample Collection

**Figure 1:**
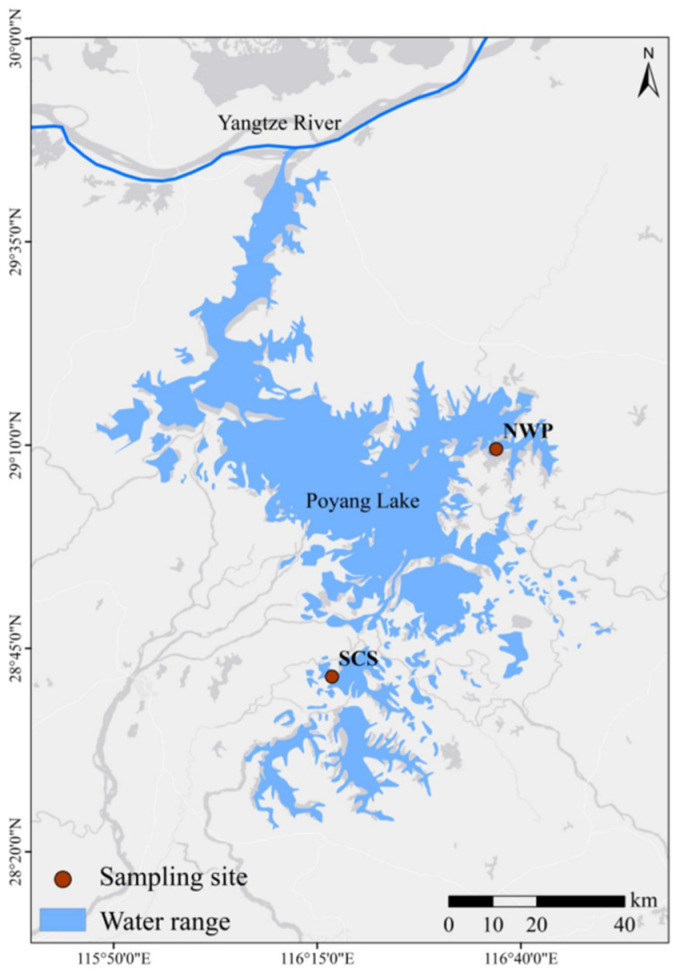
Map of sampling sites in the study area of Poyang Lake wetlands. Note: Poyang Lake (28°11′–29°51′ N, 115°49′–116°46′ E), located at the confluence of the middle and lower reaches of the Yangtze River, in northern Jiangxi Province, is the largest freshwater lake in China. The SCS (28°43′–28°48′ N, 116°11′–116°19′ E) is located in the eastern suburbs of Nanchang, adjacent to the eastern margin of Poyang Lake. This area serves as a critical conservation zone for the endangered Siberian Crane, providing a semi-natural habitat that mimics the ecological conditions of their natural wintering grounds. The NWP (28°56′–29°13′ N, 116°23′–116°44′ E), situated in Poyang County along the eastern shore of Poyang Lake, is a nationally designated wetland park that supports a diverse avian community.

Poyang Lake (28°11′–29°51′ N, 115°49′–116°46′ E), located at the confluence of the middle and lower reaches of the Yangtze River, in northern Jiangxi Province, is the largest freshwater lake in China. The region benefits from abundant sunlight, with a multi-year average temperature ranging from 16.5 to 17.8 °C. It experiences hot, rainy summers and cold, dry winters, with an annual average precipitation of 1450–1550 mm. The well-developed hydrological network and rich food resources provide an optimal habitat for wintering waterfowl, particularly migratory species [13]. This study was conducted at two strategically selected sampling sites in proximity to Poyang Lake (Figure 1): the Siberian Crane Conservation Area (SCS) within the state-owned Nanchang Wuxing Reclamation Farm and the Poyang Lake National Wetland Park (NWP). The SCS (28°43′–28°48′ N, 116°11′–116°19′ E) is located in the eastern suburbs of Nanchang, adjacent to the eastern margin of Poyang Lake. This area serves as a critical conservation zone for the endangered Siberian Crane, providing a semi-natural habitat that mimics the ecological conditions of their natural wintering grounds. The NWP (28°56′–29°13′ N, 116°23′–116°44′ E), situated in Poyang County along the eastern shore of Poyang Lake, is a nationally designated wetland park that supports a diverse avian community. This site is particularly significant for its role in harboring multiple nationally protected bird species, including the Oriental White Stork (*Ciconia boyciana*), Tundra Swan (*Cygnus columbianus*), Hooded Crane (*Grus monacha*), and Red-breasted Goose (*Branta ruficollis*).

Both study sites are separated from the main body of Poyang Lake by a levee system, yet they remain in close proximity to the natural wintering habitats of Siberian cranes. These locations share similar climatic and geographical characteristics, including comparable temperature regimes, precipitation patterns, and hydrological dynamics, which facilitate comparative analyses of microbial communities across captive and wild crane populations as well as their interactions with the soil microbial community in the wetland ecosystem.

This study design enables a comprehensive investigation of the microbial ecology in both managed conservation areas and natural wetland habitats, providing valuable insights into the ecological relationships between captive and wild Siberian Cranes and their environment.

Wild Siberian cranes overwinter in Poyang Lake (28°22′–29°45′ N, 115°47′–116°45′ E) from late October to late March (mean residence: 150 ± 7 days). During this time, individuals inhabiting artificial wetlands, such as rice paddies or lotus fields, remain until local food resources are exhausted. Wild Siberian cranes are primarily fed tubers such as lotus roots or rice.

Captive Siberian cranes were housed together in a single outdoor enclosure measuring approximately 100 m^2^. The enclosure floor consisted of simulated wetland soil, constructed from a mixture of local soil, sand, and organic matter, designed to mimic the natural wetland substrate. The enclosure included a shallow artificial pond (approximately 1 m^2^, depth 20 cm) that provided access to water for wading and drinking. Water in the artificial pond was refreshed every 48 h using clean groundwater to maintain hygiene. No additional bedding materials were provided. The cranes were fed a standardized diet consisting of corn and carrots, which were provided three times daily (morning, noon, and afternoon). Food was placed on a designated raised feeding platform to minimize direct contact with soil and reduce potential contamination of food with environmental microbes. This consistent feeding schedule and diet composition were maintained throughout the study period to ensure uniformity. No antibiotics or other medications were administered to any of the captive or wild cranes during the sampling period, based on records provided by the breeding center and field observations. Routine but limited human contact occurs during daily care activities such as feeding and cleaning.

Feces and soil samples were collected from Siberian crane habitats at two locations: SCS and NWP, following their overwintering patterns. Sampling at SCS was conducted in 15 March 2022, while at NWP, it took place in 25 March 2022. At SCS, feeding hotspots were identified using binoculars, and three major crane flocks were selected for sampling. To minimize cross-species contamination, sampling was restricted to areas where no other bird species were present within a 50 m radius during feeding. Immediately after crane departure, feces and soil samples were collected from feeding sites, identified based on crane footprints and feeding pits. Three biological replicates of feces samples were collected from each of the three wild crane flocks, with three adjacent soil samples obtained within a 5 m radius per feces deposit. This yielded a total of 18 paired samples (9 feces + 9 soil). Due to the challenges of non-invasive sampling of wild endangered cranes, the fecal samples represent population-level snapshots from each flock, and we could not control for individual identity, sex, or age. Feces samples were designated as the W group (biological triplicates: W1a–c, W2a–c, W3a–c; n = 9), and their associated soil samples as the WT group (Paired triplicates: WT1a–c, WT2a–c, WT3a–c; n = 9). For captive Siberian cranes at the National Wetland Park (NWP), an identical paired sampling protocol was implemented to maintain methodological consistency. From three separate enclosures (biological replicates), triplicate feces samples were collected per enclosure and immediately matched with adjacent enclosure soil samples (≤5 m radius, 0–10 cm depth). Feces samples from captive cranes were labeled as the Z group (Z1a–c, Z2a–c, Z3a–c; n = 9 biologically independent samples), while corresponding enclosure soil samples were categorized as the ZT group (ZT1a–c, ZT2a–c, ZT3a–c; n = 9 spatially paired controls).

To ensure sample integrity and prevent contamination, fresh feces and soil samples were collected using sterilized gloves and forceps. Any material that had come into contact with the ground was removed before transferring the samples into 10 mL sterile EP tubes.

The collection process was conducted with minimal disturbance to the crane populations. feces aliquots were flash-frozen in liquid nitrogen within 15 min of collection, soils were sieved (2 mm mesh) and stored at −80 °C until DNA extraction. All procedures complied with IUCN guidelines for research on endangered species (Approval No.: 20220315-001)

### 2.2. DNA Extraction, Amplification and High-Throughput Sequencing

Genomic DNA was extracted from feces samples (n = 18) using the feces Microbial DNA Kit (D2700, Beijing Solarbio Science & Technology Co., Ltd., Beijing, China) with bead-beating lysis (0.1 mm zirconia beads, 6 m/s for 45 s) and from soil cores (n = 18) with the Foji Soil DNA Kit (DE-05514, Chengdu Foregene Biotechnology Co., Ltd., Chengdu, China) incorporating humic acid removal steps. The extraction procedures were strictly followed according to the manufacturer’s instructions for each kit. DNA integrity was verified by 1% agarose gel electrophoresis and stored at −20 °C for subsequent use.

The V4 hypervariable regions of bacterial 16S rRNA genes were amplified using barcoded primers 515F (5′-GTGYCAGCMGCCGCGGTAA-3′) and 806R (5′-GGACTACNVGGGTWTCTAAT-3′) with Phusion High-Fidelity DNA Polymerase ((Thermo Fisher Scientific Inc., Ltd., Waltham, MA, USA)) [23]. PCR conditions included: 95 °C for 4 min (initial denaturation); 32 cycles of: 95 °C/30 s, 55 °C/30 s (annealing), 72 °C/30 s; Final extension: 72 °C/10 min. Negative controls (n = 3 per batch) were processed alongside samples. Paired-end sequencing was performed on Illumina MiSeq PE250 platform at Biomarker Technologies (Beijing, China, CAP-accredited lab.).

### 2.3. Bioinformatics Processing and Ecological Statistical Analysis

**Figure 2:**
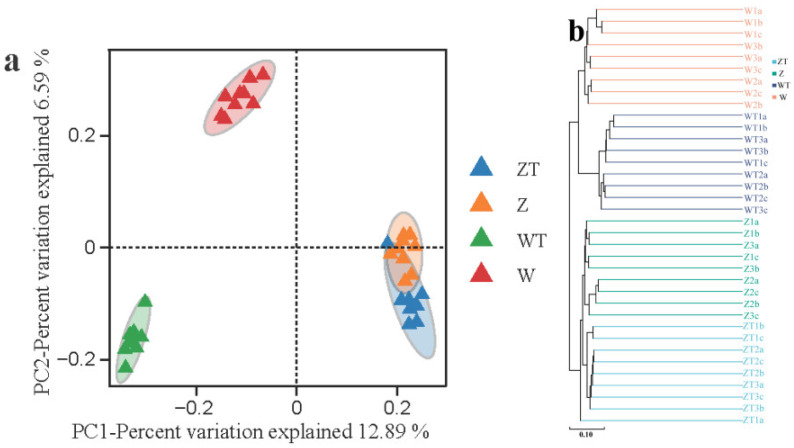
Hierarchical cluster analysis of gut microbial community structure and soil microbial community structure of Siberian cranes. (**a**) PCoA clustering analysis (weighted UniFrac: R^2^ = 0.23, *p* = 0.001). (**b**) UPGMA clustering of Bray–Curtis. Analysis was performed using the vegan package (version 2.6-4) in R (version 4.1.1).

1. Raw data quality control: Raw sequencing reads were quality-filtered using Trimmomatic-v0.33 (SLIDINGWINDOW:4:20, MINLEN:100). Adapter sequences were removed using cutadapt-v1.91.
2. Amplicon Sequence Variant (ASV) Analysis: The high-resolution ASV analysis was performed to characterize microbial community composition with single-nucleotide precision. Cleaned sequencing reads were processed using the DADA2 pipeline (v1.16.0) implemented in QIIME2 (v2020.6) to generate exact biological sequence variants (ASVs) [24].
3. Diversity Analysis: Alpha diversity indices (including observed ASVs, Chao1, ACE, Shannon, and Simpson) and species accumulation curves were calculated using R software (version 3.1.1) with the picante package (version 1.8.2) (Table 1). Beta diversity was assessed using weighted UniFrac distances to account for phylogenetic relatedness among microbial communities. Principal coordinate analysis (PCoA) and hierarchical clustering (UPGMA) were performed based on this distance matrix with significance testing via PERMANOVA (999 permutations) [25] to visualize community structure (Figure 2).
4. Taxonomic Annotation and Biomarker Analysis: Taxonomic classification of amplicon sequences was performed using the SILVA database (version 138) with a confidence threshold of 85% [26]. Sequences were assigned to taxonomic ranks using the naïve Bayesian classifier implemented in QIIME2 [25]. To minimize potential misclassifications, only sequences with a confidence score above the predefined threshold were retained for downstream analyses. The community composition of each sample was statistically analyzed at various levels (phylum, class, order, family, genus, species). QIIME2 was used to generate abundance tables at different classification levels, and R was used to visualize the community structure of each sample at various taxonomic levels. Furthermore, biomarker taxa were identified using LEfSe (Kruskal–Wallis test, LDA score > 2, and FDR-adjusted *p*-values (using the Benjamini–Hochberg procedure) for multiple testing correction) [27].
5. Cross-Domain Correlation and Network Analysis: Inter-domain microbial correlations were assessed using SparCC methods (Sparse Correlations for Compositional Data) with thresholds of 100 bootstrap iterations, 95% confidence intervals, |r| > 0.6 and *p* < 0.01. Network visualization was performed in Gephi, (v0.9.2) employing the Fruchterman-Reingold force-directed layout [28].

Raw reads with an average length of 276 bp were obtained after denoising by Illumina MiSeq sequencing. The total number of raw reads, clean reads, and the mean length of the reads were compared in all the 36 samples. The number of sequences varied from 25,391 to 170,149, and a total of 18,399 ASVs with 99% sequence similarity threshold. Detailed sequencing statistics for each individual sample are provided in Supplementary Table S1.

### 2.4. Nutritional Composition Data

Nutritional composition data for dietary items were obtained from the following sources: corn and carrot nutrient profiles from the USDA FoodData Central database (https://fdc.nal.usda.gov, accessed on 26 February 2026); lotus root and rice nutrient profiles from the China Food Composition Tables [29].

## 3. Results

### 3.1. Distinct Microbial Diversity, Composition, and Host-Environment Overlap Between Gut and Soil Microbiota

High-resolution alpha diversity metrics revealed systematic variations in microbial complexity between soil and gut compartments (*p* < 0.05, Table 1), with pronounced effects of captivity status. Soil microbiota exhibited consistently higher Ace, Chao, Simpson, and Shannon indices compared to the gut microbiota of Siberian cranes, indicating greater microbial richness and diversity in the soil environment. Among all groups, the WT (wild soil) group demonstrated the highest values for these indices, suggesting that the soil microbiota in the habitat of wild Siberian cranes harbors the most complex and diverse microbial communities.

In contrast, the gut microbiota of wild and captive Siberian cranes displayed distinct diversity patterns. Wild Siberian cranes exhibited significantly lower gut microbial richness and diversity compared to their captive counterparts, as reflected in reduced Ace, Chao, Simpson, and Shannon indices. However, an opposite trend was observed in the soil microbiota associated with their habitats, where soil from wild crane habitats showed higher diversity than that from captive environments. These findings suggest that captivity may influence gut microbial diversity while simultaneously reducing the complexity of the surrounding soil microbial communities.

PCoA based on weighted UniFrac distances demonstrated a clear separation between gut and soil microbiota across the four groups (R^2^ = 0.23, *p* = 0.001, PERMANOVA), with Axis 1 accounting for 12.89% of the variance (Figure 2a). Notably, the W group and their corresponding WT group exhibited the most pronounced separation, highlighting the greatest disparity in microbial community structures between these two groups.

Furthermore, hierarchical clustering analysis based on Bray–Curtis dissimilarity further supported this distinction, delineating four well-defined branches corresponding to the four groups (Figure 2b). All samples within the same group clustered together, forming distinct branches. The dendrogram showed that groups W and WT formed distinct major clusters from groups Z and ZT, reflecting the similarity between host gut and sympatric soil microbiota. Z group and W group formed two distinct subclusters, with a relatively large inter-cluster distance, indicating substantial divergence in gut microbial composition between captive and wild populations.

**Figure 3:**
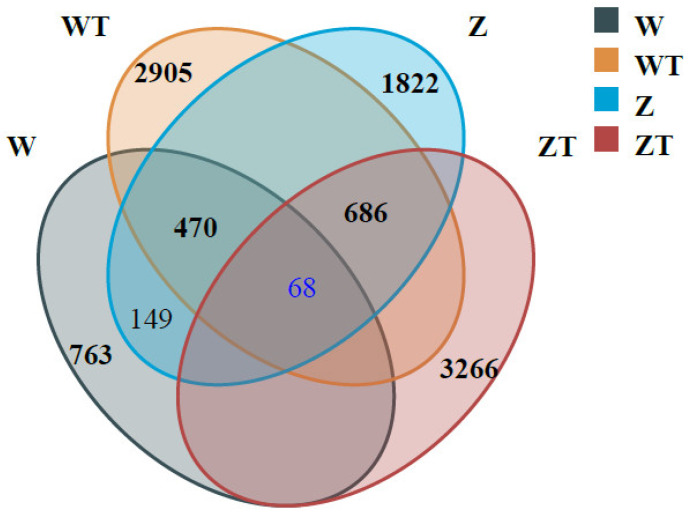
Venn diagram illustrating ASV distribution across Siberian crane gut and soil microbiota. A total of 9949 ASVs were detected across all samples. Numbers in each compartment represent: wild crane gut (W, gray, n = 1313, 763 unique ASVs), wild soil (WT, orange, n = 3479, 2905 unique ASVs), captive crane gut (Z, blue, n = 2562, 1822 unique ASVs), and captive soil (ZT, red, n = 4039, 3266 unique ASVs). Only 68 ASVs (0.7% of total) were shared among all four groups, indicating strong niche specialization. W shared 149 ASVs with Z, W shared 470 ASVs with WT, Z shared 686 ASVs with ZT.

High-resolution ASV analysis demonstrated distinct microbial niche specialization across host and environmental compartments (Figure 3). Only 68 ASVs (0.7% of total detected) were shared among all four groups (gut: W/Z; soil: WT/ZT).

W group shared 470 ASVs (35.8% of their gut microbiota) with WT group. In contrast, Z group shared a higher number of ASVs with ZT group (686 ASVs), though this represented a lower proportion (26.8%) of their gut community due to their greater overall gut microbial richness. Soil compartments (WT/ZT) accounted for 70.5% of the total unique ASVs, with ZT group displaying the highest niche specialization (37.3% unique ASVs).

### 3.2. Phylum and Genus-Level Divergence in Gut and Soil Microbiota of Captive and Wild Siberian Cranes

**Figure 4:**
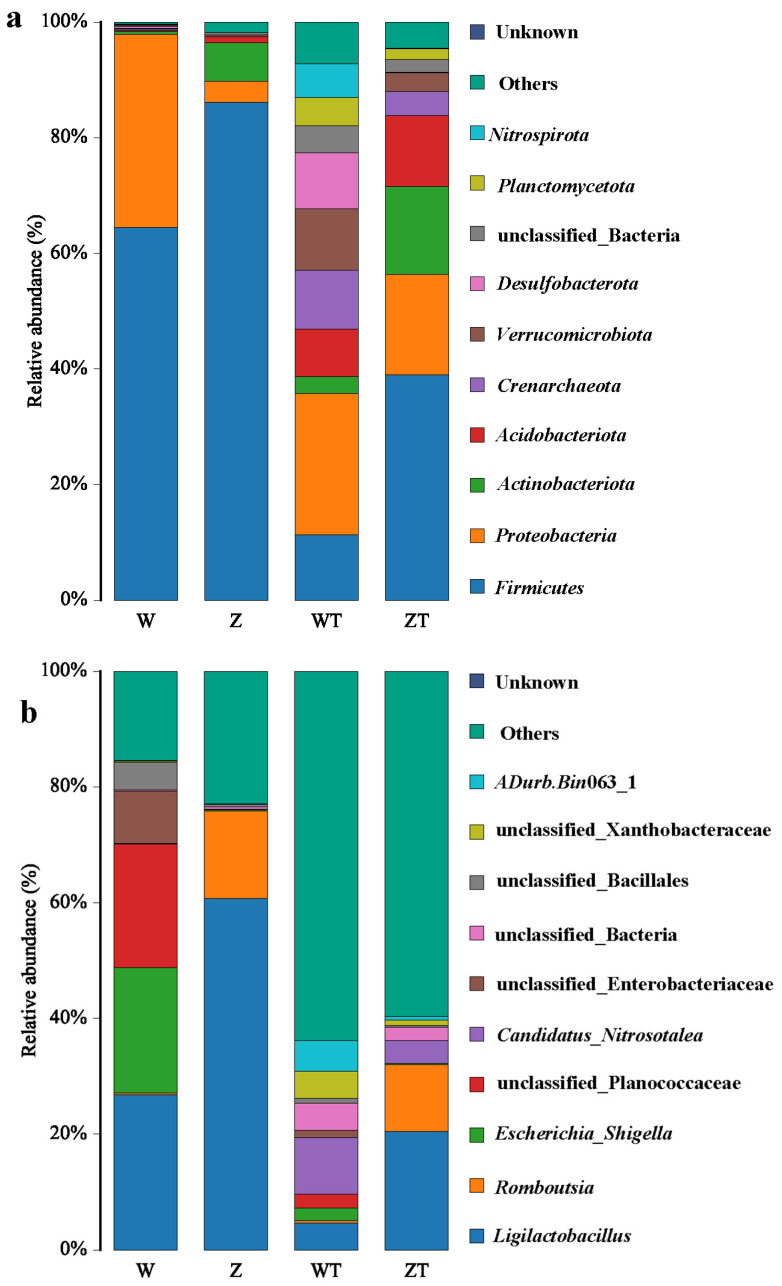
Microbial composition of gut and soil microbiota associated with captive and wild Siberian Cranes at the phylum (**a**) and genus levels (**b**). Stacked columns for the means of the individual samples from the four groups, indicating the relative abundance as a percentage of the total bacterial sequences per group. Each bar represents the mean relative abundance of microbial taxa for each group. The taxa that have relative abundance of less than 1% were combined and are referred to as “others”.

Taxonomic profiling at the phylum level revealed a distinct host–environment dichotomy in microbial community composition (Figure 4a). In the gut samples (groups W and Z) and ZT group, the dominant phyla were Firmicutes, Proteobacteria, and Actinobacteriota, with relative abundances ranging from 38.97% to 86.08%, 3.68% to 33.34%, and 0.56% to 15.14%, respectively. In contrast, WT group was dominated by Proteobacteria (24.48%), Firmicutes (11.32%), and Verrucomicrobiota (10.64%).

Notably, the relative abundance of Firmicutes was substantially higher in both gut groups (W: 64.51%; Z: 86.08%) than in the soil groups (WT: 11.32%; ZT: 38.97%). Conversely, Acidobacteriota exhibited the opposite trend, being more prevalent in soils (WT: 8.24%; ZT: 12.33%) than in gut samples (W: 0.24%; Z: 1.07%) (*p* < 0.001).

Comparing the two gut groups, W group showed a significantly higher relative abundance of Proteobacteria (W: 33.34%; Z: 3.68%) but significantly lower abundances of Firmicutes (W: 64.51%; Z: 86.08%), Actinobacteriota (W: 0.56%; Z: 6.64%), and Acidobacteriota (W: 0.24%; Z: 1.07%) relative to Z group.

At the genus level, distinct microbial signatures distinguished captive from wild cranes (Figure 4b). The most abundant genera across all samples were *Ligilactobacillus*, *Romboutsia*, and *Escherichia*–*Shigella*. However, their distributions varied markedly among groups. Specifically, *Ligilactobacillus* was substantially enriched in Z group (60.71%) compared to W group (26.85%), WT group (4.62%), and ZT group (20.42%) (*p* < 0.001 for all). Similarly, *Romboutsia* showed higher abundance in Z group (15.15%) than in the other groups (W: 0.28%; WT: 0.48%; ZT: 11.59%; *p* < 0.001). In contrast, *Escherichia*–*Shigella* was significantly more abundant in W group (21.59%) than in Z group (0.22%) and both soil groups (WT: 2.12%; ZT: 0.17%) (*p* < 0.001).

In the soil groups (WT and ZT), a large proportion of sequences could not be assigned to known genera, with unidentified genera accounting for 63.83% and 59.63% of the relative abundance in WT and ZT, respectively, reflecting the high proportion of uncharacterized microbial diversity in environmental samples.

### 3.3. Differential Microbial Abundance in Gut and Soil Microbiota Revealed by LEfSe Analysis

**Figure 5:**
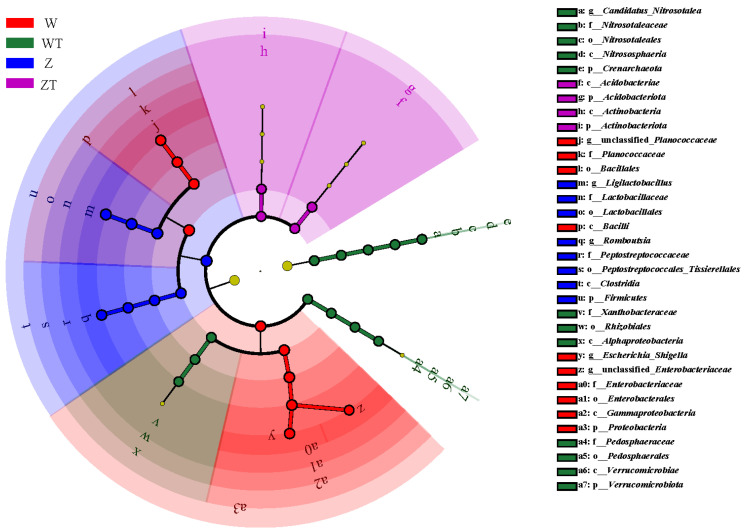
Differentially abundant taxa in gut and soil microbiota of Siberian Cranes identified by LEfSe analysis. The diagram displays taxonomic classification from the innermost to the outermost level: phylum, class, order, family, and genus. The size of the small circles corresponds to the relative abundance of species at each taxonomic level. Species with no significant differences are marked in yellow, while those showing significant differences are highlighted with color.

LEfSe analysis (LDA score > 2, *p* < 0.01) revealed 34 phylogenetically conserved biomarker taxa across five taxonomic levels, distinguishing microbial communities in the gut and soil environments (Figure 5). At the phylum level, Firmicutes predominated in the gut microbiota, while Proteobacteria was the dominant phylum in the soil microbiota, highlighting ecosystem-specific microbial composition differences.

Comparative analysis of the gut microbiota of wild and captive Siberian cranes demonstrated significant variation in their microbiota profiles. W group exhibited 10 unique biomarkers, while Z group showed 8. The gut microbiota of wild cranes was characterized by higher relative abundances of Bacilli and *Escherichia*-*Shigella*, whereas captive cranes were enriched in *Ligilactobacillus* and *Romboutsia*.

In the soil, WT group was characterized by *Candidatus Nitrosotalea*, while ZT group showed enrichment in Actinobacteriota.

### 3.4. Network-Based Insights into Gut and Soil Microbiota Interactions in Siberian Cranes

**Figure 6:**
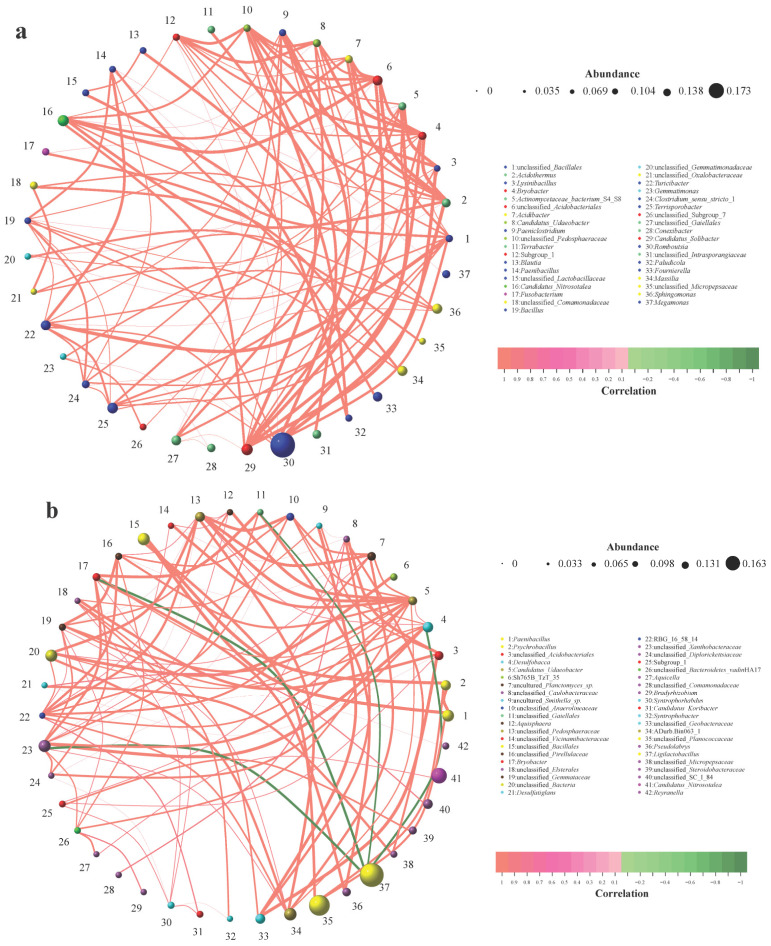

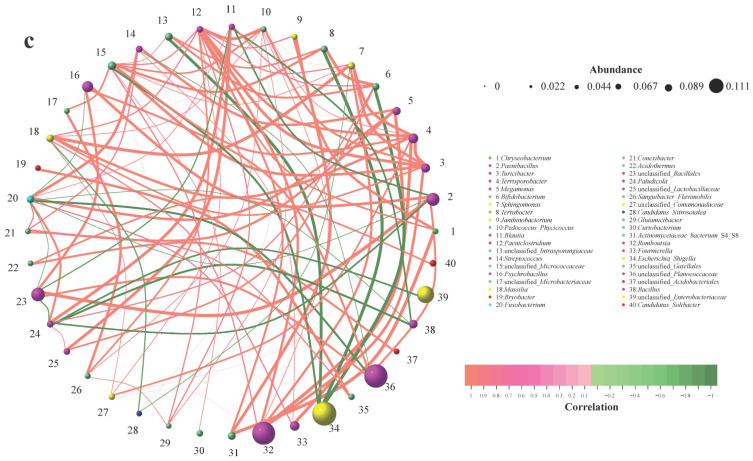
Microbial co-occurrence networks between gut and soil microbiota in Siberian cranes. (**a**) Network of captive crane feces and captive soil samples (nodes = 37, edges = 100, average degree = 5.41, modularity = 0.61). All microbial genera exhibit positive correlations. (**b**) Network of wild crane feces and wild soil samples (nodes = 42, edges = 100, average degree = 4.76, modularity = 0.52). All microbial genera exhibit positive correlations, with the exception of *Ligilactobacillus* and its associated genera. (**c**) Network between captive and wild crane feces samples (nodes = 40, edges = 100, average degree = 5.00, modularity = 0.51), showing both positive and negative correlations. Network metrics and interpretation: Nodes represent genera, with node size reflecting mean relative abundance. Edges represent significant correlations (|r| > 0.6, *p* < 0.01, SparCC), with edge thickness indicating correlation strength. Edge colors denote correlation type: red for positive, green for negative. Node count reflects network scale, edge count reflects connectivity density, average degree measures mean connections per node, and modularity (>0.4 indicates significant modular structure) reflects network compartmentalization and stability.

From the microbial network relationship graph between captive Siberian crane and the soil of their habitat, it is observable that all the bacterial genera exhibit positive correlations (Figure 6a). Among them, the genera that have the most intimate interaction with other genera are unclassified_*Bacillales*, *Acidothermus*, unclassified_*Acidobacteriales*, unclassified_*Pedosphaeraceae*, *Subgroup_1*, *Bacillus*, *Turicibacter*, *Candidatus_Solibacter* and *Romboutsia*. These genera are concurrently significantly and positively correlated with 9 other genera (*p* < 0.01).

From the microbial network relationship graph between wild Siberian crane and the soil in the same area, it can be noted that all other genera are positively correlated, except for *Ligilactobacillus* and its associated genera, which present negative correlations (Figure 6b). Among them, the genus with the most intimate interaction with other genera is *Candidatus_Udaeobacter*, which is significantly and positively correlated with 13 other genera (*p* < 0.01). Additionally, unclassified_*Xanthobacteraceae* also has a close interaction with other genera and is significantly correlated with 12 other genera (*p* < 0.01). Furthermore, unclassified_*Pedosphaeraceae14* is significantly correlated with 11 other genera (*p* < 0.01).

From the microbial network relationship graph between captive and wild Siberian crane, it can be discerned that there are both positive and negative correlations among the bacterial genera (Figure 6c). Among them, the genera with the most intimate interaction with other genera are *Paeniclostridium* and unclassified_*Micrococcaceae*. *Paeniclostridium* was significantly and positively correlated with 12 other genera (*p* < 0.01), while unclassified_*Micrococcaceae* was significantly and positively correlated with 11 other genera (*p* < 0.01) and significantly negatively correlated with one genus (*p* < 0.01). Moreover, *Escherichia_Shigella* is solely significantly and negatively correlated with other genera (*p* < 0.01).

## 4. Discussion

The significantly higher microbial diversity and richness observed in the soil of Siberian crane habitats compared to their intestinal microbiota underscore the distinct environmental conditions that govern microbial community assembly. The soil environment, characterized by aerobic and weakly alkaline conditions, contrasts sharply with the anaerobic and weakly acidic intestinal environment of the cranes [21]. These stark physicochemical differences impose strong selective pressures on microbial colonization, leading to distinct community structures in each habitat. Oxygen availability, pH gradients, and nutrient composition likely drive these ecological divergences, shaping microbial interactions and functional adaptations.

A key finding of this study is the higher gut microbial richness and diversity in captive Siberian cranes relative to their wild counterparts. The finding is consistent with patterns observed in red-crowned cranes (*Grus japonensis*) [30] and common cranes (*Grus grus*), suggesting that similar ecological changes associated with captivity may influence gut microbiota across crane taxa. However, given the limited sample size in each study, further research with larger sample sizes is needed to confirm this pattern. The finding aligns with the Dietary Filtering Hypothesis [31,32]. Standardized captive diets may relax niche competition, supporting greater microbial richness compared to specialized wild foraging. Captive cranes were fed corn (~64% starch, ~74% carbohydrates) and carrots (~4.7% sugars, ~2.8% fiber) three times daily. This combination provides diverse carbohydrate substrates (resistant starch, cellulose, hemicellulose, pectins), creating multiple metabolic niches that support broader microbial taxa [33]. Consistent food availability may also reduce competitive exclusion [32]. In contrast, wild cranes forage on lotus roots (moderate starch: ~11%; fiber: ~4.9%) and rice stubble. While rice grains are starch-dense (~77%), the cellulose-rich rice straw (35–40% cellulose) dominates their intake, presenting a structurally complex but compositionally narrower substrate. Cellulose digestion requires specialized cellulolytic bacteria [34], potentially constraining diversity by favoring fiber-degrading specialists. Seasonal and spatial heterogeneity of wild resources may further filter microbial communities [35].

The degree of similarity between the gut microbiota of Siberian cranes and their habitat soil underscores potential microbial exchange. Specifically, 26.77% of total gut ASVs in captive cranes and 35.80% in wild cranes overlapped with their respective soil microbiota. In contrast, only 16.08% of gut ASVs in adult black-billed gulls (*Larus saundersi*) were shared with soil [36], indicating stronger microbial associations between Siberian cranes and their habitat. This disparity may be attributed to foraging behavior—Siberian cranes feed by excavating plant roots and stems in shallow waters, increasing direct soil contact, whereas black-billed gulls primarily consume aquatic prey, limiting soil interaction. These findings align with the habitat filtering hypothesis, which posits that environmental constraints shape microbial community assembly [37]. The minimal ASV overlap (149) between wild and captive cranes suggests strong host-environment physiological filters, such as gut pH, bile acids, and immune responses, which regulate microbial colonization and create a barrier effect [38]. However, the greater ASV sharing between captive cranes and their habitat soil (Z-ZT = 686) compared to wild cranes and their soil (W-WT = 470) suggests that captivity may weaken microbial transmission barriers. This could result from altered diets and reduced exposure to diverse environmental microbial sources, leading to a captivity gradient [39]. The high number of unique ASVs in captive soil (ZT = 3266) likely reflects anthropogenic selection pressures, including fertilizer use and reduced plant diversity, which reshape soil microbial composition. These changes may indirectly influence gut microbiota diversity in captive birds by modifying environmental microbial pools [40].

Our findings contribute to the growing evidence that avian gut microbiota assembly is shaped by factors across multiple spatial and temporal scales. While our study highlights broad contrasts between captive and wild environments, recent research emphasizes that fine-scale variation within populations can be equally substantial. For instance, a study showed that microhabitat heterogeneity structures microbiota in European pied flycatchers at a local scale [41]. Similarly, studies on finches have demonstrated strong host-specific signatures in finches even under controlled conditions, underscoring host identity as a key variable [42], while others have revealed that seasonal effects interact with environmental and individual-level factors rather than acting alone [43]. Most directly relevant, foraging environment shapes gut microbiota in cranes within a single wetland system [44], corroborating our observation that local habitat conditions, such as soil microbial communities, may contribute to microbial assembly. Collectively, these studies suggest that the patterns we observed arise from a complex interplay between captivity, seasonal migration, and fine-scale habitat variation, rather than any single factor in isolation. This interpretation, however, remains speculative and requires direct experimental testing.

This study identified Firmicutes, Proteobacteria, and Actinobacteriota as the dominant bacterial phyla in the intestinal microbiota of both captive and wild Siberian cranes, consistent with findings from wintering Siberian cranes in Poyang Lake [17]. Among these, Firmicutes was the most prevalent, which aligns with studies on the gut microbiota of other crane species, such as the white-naped crane (*Grus vipio*) [45] and the red-crowned crane (*Grus japonensis*) [46]. Firmicutes play a crucial role in the breakdown of complex carbohydrates, fatty acids, and polysaccharides in the gut, facilitating host energy metabolism [34]. Notably, the relative abundance of Firmicutes in wild Siberian cranes (W group) was 86.08%, significantly higher than in captive Siberian cranes (Z group) at 64.51%. This difference may be attributed to the higher sugar content in the natural diet of wild cranes, which could promote Firmicutes proliferation. Additionally, captive conditions may lead to a relaxation of environmental microbial filters, reducing the number of discriminatory taxa by approximately 67% compared to wild-type soil microbiota. This reduction could weaken microbial transmission barriers and homogenize gut microbial niches in captivity [47].

At the genus level, *Ligilactobacillus* and *Romboutsia* exhibited the highest relative abundances in captive Siberian cranes, whereas *Escherichia*-*Shigella* was most abundant in wild individuals. LEfSe analysis further confirmed that *Ligilactobacillus*, and *Romboutsia* were significantly enriched in captive Siberian cranes. *Ligilactobacillus* is known to ferment sugars and produce lactic acid [48], its enrichment in captive cranes may reflect dietary starch and sugar content, though functional consequences for host health require direct investigation. Similarly, *Romboutsia* plays a key role in host well-being, with its higher abundance potentially enhancing survival [49]. In contrast, *Escherichia*-*Shigella* was more abundant in wild cranes. While some members of this genus can be pathogenic, this pattern may reflect environmental exposure rather than disease risk [50].

We acknowledge several limitations. The small sample size of cranes (n = 3) limits statistical power and generalizability. The cross-sectional design captures only a snapshot, failing to reflect temporal dynamics. 16S rRNA sequencing provides taxonomic profiles without functional verification, and uncontrolled host variables (age, sex) may confound comparisons. LEfSe can be unstable in small or sparse datasets and may inflate Type I error rates [51]. Future research should employ metagenomic/metabolomic analyses to elucidate microbial functions, longitudinal monitoring across overwintering periods to capture dynamic changes, and larger-scale individual-level sampling to account for host heterogeneity. Addressing these limitations will deepen our understanding of captivity-induced microbiota shifts and their implications for crane conservation.

## 5. Conclusions

This study provides a comparative analysis of the gut microbiota of captive and wild Siberian cranes and their associations with soil microbial communities in the Poyang Lake Wetlands. By integrating compositional profiling with co-occurrence network analysis, we observed distinct microbial community structures between captive and wild individuals, as well as between gut and soil environments. Notably, we observed a distinct microbial network structure in wild Siberian cranes, in contrast to the associations observed in captivity. A key finding is the significant negative correlation between the gen and the gut microbiota of wild cranes, which is an interaction absent in captive environments. Additionally, the pattern of exclusively positive correlations between captive cranes and their soil microbiota differs from the assumption that captivity universally disrupts host-microbiota interactions. Instead, our results suggest that captivity may be associated with changes in microbial interactions and gut microbiota functions. However, due to the limited sample size, these findings should be interpreted as preliminary and require further validation.

## Figures and Tables

**Table 1:** Alpha diversity indices of gut and soil microbiota.

Sample	ASV	ACE	Chao	Simpson	Shannon
W	220 ± 38 ^c^	229 ± 13 ^c^	239 ± 13 ^c^	0.794 ± 0.010 ^c^	3.213 ± 0.111 ^d^
Z	415 ± 93 ^b^	418 ± 31 ^b^	421 ± 30 ^b^	0.821 ± 0.012 ^c^	3.965 ± 0.091 ^c^
WT	734 ± 134 ^a^	739 ± 45 ^a^	743 ± 44 ^a^	0.989 ± 0.002 ^a^	8.027 ± 0.075 ^a^
ZT	676 ± 241 ^a^	678 ± 80 ^a^	681 ± 80 ^a^	0.949 ± 0.015 ^b^	6.916 ± 0.278 ^b^

Data are presented as mean ± SD. Superscript letters (a, b, c, d) denote statistically homogeneous subgroups based on Tukey’s HSD post hoc test (α = 0.05) following a significant one-way ANOVA. ACE/Chao1 reflect microbial richness, Simpson/Shannon reflect microbial diversity and evenness. W/Z: wild/captive cranes; WT/ZT: their associated soils.

## Data Availability

Data will be made available on request.

## Supplementary Materials

The following supporting information can be downloaded at: https://www.mdpi.com/article/10.3390/ani16060894/s1, Table S1: Information of samples by 16S rRNA amplicon sequencing.

## References

[1] Liu H., Liao C., Wu L., Tang J., Chen J., Lei C., Zheng L., Zhang C., Liu Y., Xavier J. (2022). Ecological dynamics of the gut microbiome in response to dietary fiber. ISME J..

[2] Lindsay E.C., Metcalfe N.B., Llewellyn M.S. (2020). The potential role of the gut microbiota in shaping host energetics and metabolic rate. J. Anim. Ecol..

[3] Sun F., Chen J., Liu K., Tang M., Yang Y. (2022). The avian gut microbiota: Diversity, influencing factors, and future directions. Front. Microbiol..

[4] Li C., Liu Y., Gong M., Zheng C., Zhang C., Li H., Wen W., Wang Y., Liu G. (2021). Diet-induced microbiome shifts of sympatric overwintering birds. Appl. Microbiol. Biotechnol..

[5] Li L., Ye J., Yu M., Jiang J., Guo X., Yu W., Rong K. (2025). Dynamic changes in the avian gut microbiome in response to diverse lifestyles. Appl. Microbiol. Biotechnol..

[6] Hill S.C., François S., Thézé J., Smith A.L., Simmonds P., Perrins C.M., van der Hoek L., Pybus O.G. (2023). Impact of host age on viral and bacterial communities in a waterbird population. ISME J..

[7] Eliades S.J., Brown J.C., Colston T.J., Fisher R.N., Niukula J.B., Gray K., Siler C.D. (2021). Gut microbial ecology of the critically endangered Fijian crested iguana (*Brachylophus vitiensis*): Effects of captivity status and host reintroduction on endogenous microbiomes. Ecol. Evol..

[8] Tang S., Li Y., Huang C., Yan S., Li Y., Chen Z., Wu Z. (2022). Comparison of gut microbiota diversity between captive and wild tokay gecko (*Gekko gecko*). Front. Microbiol..

[9] Wang W., Wang Y., Chen Q., Ding H. (2023). Effects of diet shift on the gut microbiota of the critically endangered Siberian crane. Avian Res..

[10] Heijtz R.D., Wang S.G., Anura F., Qian Y., Bjorkholm B., Samuelsson A., Pettersson S. (2011). Normal gut microbiota modulates brain development and behavior. Proc. Natl. Acad. Sci. USA.

[11] Kohl K.D., Brun A., Magallanes M., Brinkerhoff J., Laspiur A., Acosta J.C., Bordenstein S. (2016). Gut microbial ecology of lizards: Insights into diversity in the wild, effects of captivity, variation across gut regions and transmission. Mol. Ecol..

[12] Zheng Z.X. (2002). Birds of the World.

[13] Shao M.Q. (2021). Bird Diversity and Ecological Habits of Poyang Lake and Five River Systems.

[14] Hou J., Li L., Wang Y., Wang W., Zhan H., Dai N., Lu P. (2021). Influences of submerged plant collapse on diet composition, breadth, and overlap among four crane species at Poyang Lake, China. Front. Zool..

[15] Shao M., Guo H., Jiang J. (2014). Population sizes and group characteristics of Siberian Crane (*Leucogeranus leucogeranus*) and Hooded Crane (*Grus monacha*) in Poyang Lake wetlands. Zool. Res..

[16] Wang Y., Long Z., Zhang Y., Li X., Zhang X., Su H. (2023). Host genetic background rather than diet-induced gut microbiota shifts of sympatric black-necked crane, common crane and bar-headed goose. Front. Microbiol..

[17] Lai Z., Sheng Y., Xiao L., Yang H., Yang W., Jian M. (2023). Gut microbiota structure and function of Siberian crane (*Grus leucogeranus*) overwintering in Poyang Lake. Acta Microbiol. Sin..

[18] Fierer N., Wood S., Mesquita C.P.B.D. (2021). How microbes can, and cannot, be used to assess soil health. Soil Biol. Biochem..

[19] Delgado-Baquerizo M., Oliverio A.M., Brewer T.E., Benavent-Gonzalez A., Eldridge D.J., Bardgett R.D., Maestre F.T., Singh B.K., Fiere N. (2018). A global atlas of the dominant bacteria found in soil. Science.

[20] Youngblut N.D., Reischer G.H., Walters W., Schuster N., Walzer C., Stalder G., Ley R.E., Farnleitner A.H. (2019). Host diet and evolutionary history explain different aspects of gut microbiome diversity among vertebrate clades. Nat. Commun..

[21] Berg G., Rybakova D., Fischer D., Cernava T., Vergès M.C.C., Charles T., Chen X., Cocolin L., Eversole K., Corral G.H. (2020). Microbiome definition re-visited: Old concepts and new challenges. Microbiome.

[22] Zhou D., Bai Z., Zhang H., Li N., Bai Z., Cheng F., Lu Z. (2018). Soil is a key factor influencing gut microbiota and its effect is comparable to that exerted by diet for mice. F1000Research.

[23] Dennis K.L., Wang Y.W., Blatner N.R., Wang S.Y., Saadalla A., Trudeau E., Roers A., Weaver C.T., Lee J.J., Gilbert J.A. (2013). Adenomatous polyps are driven by microbe-instigated focal inflammation and are controlled by IL-10-producing T cells. Cancer Res..

[24] Callahan B.J., McMurdie P.J., Rosen M.J., Han A.W., Johnson A.J.A., Holmes S.P. (2016). DADA2: High-resolution sample inference from Illumina amplicon data. Nat. Methods.

[25] Bolyen E., Rideout J.R., Dillon M.R., Bokulich N.A., Abnet C.C., Al-Ghalith G.A., Alexander H., Alm E.J., Arumugam M., Asnicar F. (2019). Reproducible, interactive, scalable and extensible microbiome data science using QIIME 2. Nat. Biotechnol..

[26] Murali A., Bhargava A., Wright E.S. (2018). IDTAXA: A novel approach for accurate taxonomic classification of microbiome sequences. Microbiome.

[27] Segata N., Izard J., Waldron L., Gevers D., Miropolsky L., Garrett W.S., Huttenhower C. (2011). Metagenomic biomarker discovery and explanation. Genome Biol..

[28] Weiss S., Van Treuren W., Lozupone C., Faust K., Friedman J., Deng Y., Xia L.C., Xu Z.Z., Ursell L., Alm E.J. (2016). Correlation detection strategies in microbial data sets vary widely in sensitivity and precision. ISME J..

[29] Yang Y.X. (2018). China Food Composition Tables.

[30] Xie Y., Xia P., Wang P., Yu H., Giesy J.P., Zhang Y., Zhang X. (2016). Effects of captivity and artificial breeding on microbiota in feces of the red-crowned crane (*Grus japonensis*). Sci. Rep..

[31] Amato K.R., Yeoman C.J., Kent A., Righin N., Carbonero F., Estrada A., Gaskins H.R., Stumpf R.M., Yildirim S., Torralba M. (2013). Habitat degradation impacts black howler monkey (*Alouatta pigra*) gastrointestinal microbiomes. ISME J..

[32] Clayton J.B., Vangay P., Huang H., Ward T., Hillmann B.M., Al-Ghalith G.A., Travis D.A., Long H.T., Van T.B., Van M.V. (2016). Captivity humanizes the primate microbiome. Proc. Natl. Acad. Sci. USA.

[33] Haidar-Ahmad N., Manigat F.O., Silue N., Pontier S.M., Campbell-Valois F.X. (2023). A tale about Shigella: Evolution, plasmid, and virulence. Microorganisms.

[34] Flint H.J., Bayer E.A., Rincon M.T., Lamed R., White B.A. (2008). Polysaccharide utilization by gut bacteria: Potential for new insights from genomic analysis. Nat. Rev. Microbiol..

[35] McKenzie V.J., Song S.J., Delsuc F., Prest T.L., Oliverio A.M., Korpita T.M., Alexiev A., Amato K.R., Metcalf J.L., Kowalewski M. (2017). The effects of captivity on the mammalian gut microbiome. Integr. Comp. Biol..

[36] Chen J., Xu Y., Liu Y., Liu K., Wu Y., Zhang Y., Yang Y. (2022). Gut microbiota analysis and gene function prediction among young and adult *Larus saundersi* with habitat soil in the Yellow River Delta. Bioresour. Technol. Rep..

[37] Justel-Díez M., Delgadillo-Nuño E., Gutiérrez-Barral A., García-Otero P., Alonso-Barciela I., Pereira-Villanueva P., Álvarez-Salgado X.A., Velando A., Teira E., Fernández E. (2023). Inputs of seabird guano alter microbial growth, community composition and the phytoplankton–bacterial interactions in a coastal system. Environ. Microbiol..

[38] Li W., Hang S., Fang Y., Bae S., Zhang Y., Zhang M., Wang G., McCurry M.D., Bae M., Paik D. (2021). A bacterial bile acid metabolite modulates Treg activity through the nuclear hormone receptor NR4A1. Cell Host Microbe.

[39] Wu H., Wu N., Liu X., Zhang L., Zhao D. (2024). Diet drives gut bacterial diversity of wild and semi-captive common cranes (*Grus grus*). Animals.

[40] Ai C., Zhang S., Zhang X., Guo D., Zhou W., Huang S. (2018). Distinct responses of soil bacterial and fungal communities to changes in fertilization regime and crop rotation. Geoderma.

[41] Videvall E., Ruiz-Limón P., Martínez-Padilla J., Moreno-Indias I., Canal D., Muriel J. (2026). Fine-scale variation in the gut microbiome of the European pied flycatcher (*Ficedula hypoleuca*) in central Spain. Ardeola.

[42] Maraci Ö., Antonatou-Papaioannou A., Jünemann S., Castillo-Gutiérrez O., Busche T., Kalinowski J., Caspers B.A. (2021). The gut microbial composition is species-specific and individual-specific in two species of estrildid finches, the Bengalese finch and the zebra finch. Front. Microbiol..

[43] Liukkonen M., Muriel J., Martínez-Padilla J., Nord A., Pakanen V.M., Rosivall B., Tilgar V., van Oers K., Grond K., Ruuskanen S. (2024). Seasonal and environmental factors contribute to the variation in the gut microbiome: A large-scale study of a small bird. J. Anim. Ecol..

[44] Gao X., Liu Y., Zhou B., Yu J., Li L., Wu Q., Wang J., Shang S. (2026). Foraging environment shapes the gut microbiota of two crane species in the Yellow River Delta wetland. Diversity.

[45] Zhang N., Zhou L., Yang Z., Gu J. (2021). Effects of food changes on intestinal bacterial diversity of wintering hooded cranes (*Grus monacha*). Animals.

[46] Zhao X., Ye W., Xu W., Xu N., Zheng J., Chen R., Liu H. (2022). Changes in the diversity and composition of gut microbiota of Red-Crowned Cranes (*Grus japonensis*) after avian influenza vaccine and anthelmintic treatment. Animals.

[47] Diaz J., Reese A.T. (2021). Possibilities and limits for using the gut microbiome to improve captive animal health. Anim. Microbiome.

[48] Pereira G.V.D.M., Coelho B.D.O., Júnior A.I.M., Thomaz-Soccol V., Soccol C.R. (2018). How to select a probiotic? A review and update of methods and criteria. Biotechnol. Adv..

[49] Gerritsen J., Hornung B., Ritari J., Paulin L., Rijkers G.T., Schaap P.J., de Vos W.M., Smidt H. (2019). A comparative and functional genomics analysis of the genus *Romboutsia* provides insight into adaptation to an intestinal lifestyle. BioRxiv.

[50] Davidson G.L., Wiley N., Cooke A.C., Johnson C.N., Fouhy F., Reichert M.S., de la Hera I., Crane J.M.S., Kulahci I.G., Ross R.P. (2020). Diet induces parallel changes to the gut microbiota and problem solving performance in a wild bird. Sci. Rep..

[51] Pang S. (2025). Improving linear discriminant analysis effect size analysis to enhance its reliability in small sample sizes. Turk. J. Pediatr..

